# A natural antisense transcript of the *Petunia hybrida Sho* gene suggests a role for an antisense mechanism in cytokinin regulation

**DOI:** 10.1111/j.1365-313X.2007.03309.x

**Published:** 2007-12

**Authors:** Elena Zubko, Peter Meyer

**Affiliations:** Centre for Plant Sciences, University of Leeds Leeds LS2 9JT, UK

**Keywords:** natural antisense transcript, cytokinin, small RNA, RNA turnover, plant hormone, *Petunia hybrida*

## Abstract

The *Sho* gene from *Petunia hybrida* encodes an enzyme responsible for the synthesis of plant cytokinins. The 3′ region of the *Sho* gene contains a promoter in the opposite orientation that produces a partially overlapping antisense transcript. Although *Sho* expression varies significantly in individual cell types, the sense and antisense transcript levels maintain a stable ratio in most tissue types. In reporter lines for the antisense promoter, we observed a change in antisense promoter activity in newly formed tissue that had been induced by prolonged culture on cytokinins or following decapitation. We interpret these data as a reflection of tissue-specific threshold levels for activation of the antisense transcript. In all tissue types tested, we detect a pool of antisense RNA of approximately 35 nt, which derives from the region where *Sho* sense and antisense transcripts overlap. We detect a second pool of putative dsRNA breakdown products of approximately 24 nt in all tissues tested, except roots, which are the main source of cytokinin synthesis. Our data suggest that antisense transcription can be activated in a tissue-specific manner to adjust local cytokinin synthesis via degradation of *Sho* dsRNA. We therefore propose that, in addition to cytokinin transport and inactivation, regulation of local cytokinin synthesis via antisense transcription represents yet another device for the complex control of local cytokinin levels in plants.

## Introduction

The expression of antisense constructs is a well-established procedure to reduce the activity of individual genes in plants (Hamilton *et al.*, 1990). It may be expected that this effect is based on an intrinsic natural mechanism that would allow plants to regulate the expression of endogenous genes via antisense expression. So far, natural occurring antisense transcripts have been predominantly documented in animal systems and prokaryotes (Terryn and Rouze, 2000). Originally described in prokaryotes (Wagner and Simons, 1994), natural antisense transcripts (NATs) have been detected in various eukaryotes, including slimemolds, insects, amphibians, birds and mammals, including humans (Vanhee-Brossollet *et al.*, 1995). While the functions of the affected ORFs differ, many are involved in proliferation control and hormonal response. Some NATs, such as c-myc and bFGF, are conserved among different species, suggesting that NAT-based regulation systems have been maintained throughout evolution.

In plants, there are few reports regarding NATs (Terryn and Rouze, 2000). The Arabidopsis kinase-like (AKL) gene has a non-coding antisense transcript that derives from alternative splicing of the inverse strand (Terryn *et al.*, 1998). A *Brassica oleracea* antisense SRK transcript has been shown to inhibit translation of a sense transcript *in vitro* (Cock *et al.*, 1997). Differentially expressed NATs associated with other members of the SRK family have also been described in maize (Ansaldi *et al.*, 2000). Potential NAT-based regulation systems in plants may be especially relevant for genes involved in developmental control or adaptive responses to changing environmental conditions. This assumption has recently been supported by the detection of NAT-specific small RNAs in plants that appear when antisense transcription is induced by salt stress (Borsani *et al.*, 2005) or following pathogen attack (Katiyar-Agarwal *et al.*, 2006).

Cytokinins (CKs) are a group of essential plant hormones that are involved in shoot meristem and leaf formation, cell division, senescence and chloroplast biogenesis (Mok *et al.*, 2000). CKs are synthesized in the root tip, and it has long been thought that root to shoot transport via the xylem is the only route for CK supply. However, cytokinins are also synthesized in aerial parts, especially in tissues rich in dividing cells (Nordstrom *et al.*, 2004). Local CK levels are therefore controlled by a complex set of mechanisms that regulate CK synthesis, CK transport (Burkle *et al.*, 2003), reversible and irreversible CK inactivation via *N*- or *O*-glycosylation (Kaminek *et al.*, 1997), and CK oxidation (Schmulling *et al.*, 2003).

Our group has recently identified a CK-producing gene (*Sho*) in *Petunia hybrida* (Zubko *et al.*, 2002). Enhanced *Sho* expression induces a significant increase in cytokinins, which mainly consist of *N*^2^-(Δ^2^-Isopentenyl) adenine (2iP) derivatives. The observation that the *Sho* gene contains an antisense ORF that partly overlaps with the ORF of the *Sho* sense transcript prompted us to examine the potential presence of a NAT system that represents an additional control system for the regulation of local cytokinin levels.

## Results

### The Sho locus contains an antisense-specific promoter

**Figure 1:**
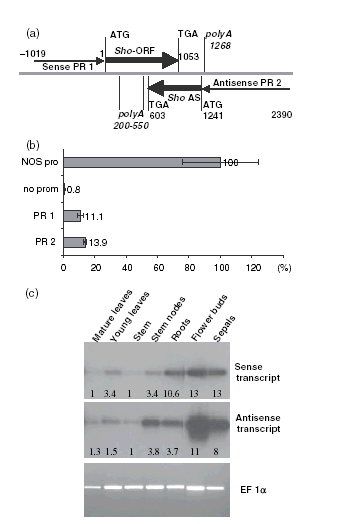
The *Sho* locus contains a partially overlapping antisense transcript. (a) Schematic map of the *Sho* locus. The open reading frame encoding the SHO protein overlaps with an antisense open reading frame, *Sho* AS. Nucleotide positions are shown by numbers adjusted to position +1 at the ATG of the *Sho* ORF. The sense transcript is polyadenylated at position 1268, while antisense transcripts have a range of polyadenylation sites within the region 200–550. (b) The sense promoter PR1 and the antisense promoter PR2 show comparable expression levels in transient assays, which are about one magnitude lower than that of the nopaline synthase (NOS) promoter. (c) RT-PCR with strand-specific primers for the *Sho* sense and antisense transcript, respectively, for mature plants. Values relative to stem-specific values are shown. Sense and antisense trancript levels vary in different tissues but maintain similar ratios to each other in most tissues.

Sequence analysis of the *Sho* locus revealed that it contained a second ORF in the opposite orientation to the ORF that encodes the SHO protein. The 1053 bp *Sho* ORF and the 639 bp ORF of the *Sho* antisense transcript share a 450 bp overlap (Figure 1a). The antisense ORF does not match any known proteins in the database. It is unclear whether it fulfils any role in the regulation of CK synthesis. To test whether the two ORFs reflect transcripts produced by the activity of two converging promoters from each end of the *Sho* region, we isolated the two putative promoter regions, comprising a 2185 bp fragment upstream of the *Sho* ORF and a 1149 bp fragment upstream of the *Sho* antisense ORF. Each fragment was inserted upstream of a promoterless GUS marker gene and a nopaline synthese (NOS) polyA region. The resulting constructs PR1–GUS and PR2–GUS were transferred into tobacco protoplasts, with a NOS–GUS construct and a promoterless GUS construct as controls. Transient expression assays revealed that both promoters were active at a comparable level, which was about one magnitude lower than the NOS promoter activity (Figure 1b). A test of a series of promoter deletion constructs in transient expression assays demonstrated the robustness of the *Sho* antisense promoter, as it still retained 20% activity even after the removal of all putative CAAT and TATA sites (Figure S1).

To test whether and where sense and antisense transcripts of the *Sho* region are produced in *Petunia hybrida*, we designed sense- and antisense-specific primers that contained *Sho* homologous sequences at their 3′ end preceded by novel primer sequences. These primers were used in strand-specific RT-PCR reactions using RNA isolated from various tissues. When we examined transcripts in whole seedlings, using similar PCR cycle conditions, antisense-specific PCR fragments were less abundant than sense-specific PCR products. However, although we used a reference gene to standardize the cDNA reactions, it is necessary to exercise caution regarding any conclusions drawn about the quantitative representation of the two transcripts in the same sample, because the strand-specific primers may differ in annealing efficiency. Our assay provides a good measure of the relative presence of each transcript in the various tissues. In most tissues, increased levels of *Sho* sense transcript are matched by a comparable increase in *Sho* antisense transcript levels. In roots and young leaves, sense transcripts are slightly increased relative to antisense transcript levels (Figure 1c). Mapping of the polyadenylation sites of the two transcripts by RT-PCR using an oligo(dT) primer and a strand-specific primer, revealed a single polyadenylation site for the *Sho* sense transcript and a variable polyadenylation region for the antisense transcript (Figure S2).

### Changes in cell type can affect the activity of the Sho antisense promoter

**Figure 2:**
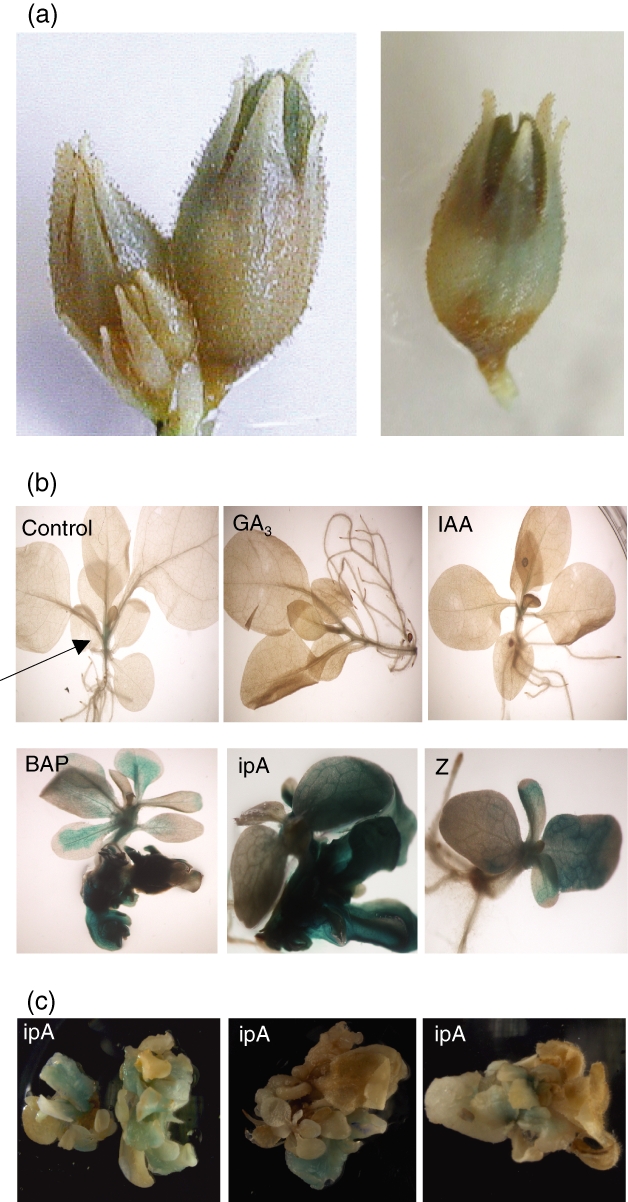
Local activity of the *Sho* antisense promoter in transformants. (a) Activity of a PR2–GUS reporter construct in flower buds. (b) Antisense promoter activity in tissue exposed to hormones. An untreated seedling displays antisense promoter activity in the stem region, indicated by the arrow. In seedlings cultivated for 21 days on GA_3_ or IAA, the expression pattern does not change, while exposure to the cytokinins BAP, IpA and zeatin (Z) induces enhanced GUS activity in a PR2–GUS reporter line. The root tissue in particular, which has converted into undifferentiated tissue, is intensely stained. Enhanced antisense promoter activity is also detectable in the stem and in the vein system, from which it spreads into the leaf area. (c) Three PR2 reporter lines that show a similar but less strong response as the lines shown in Figure 2b, to cytokinin-mediated cell type changes. After 5 weeks of IpA application, most tissues have reorganized into callus-like structures with enhanced GUS activity, especially in the basal regions.

**Figure 3:**
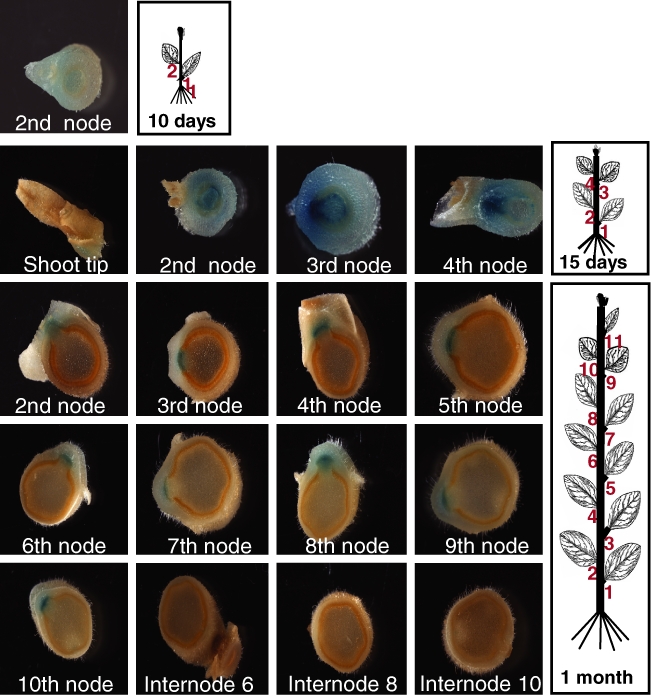
Node-specific activity of the *Sho* antisense promoter. In transverse stem sections of 10-, 15- and 30-day-old PR2–GUS transformants, the antisense promoter shows an enhanced activity in nodes around the base of axillary buds, whose development is inhibited due to apical dominance. This effect is particularly strong in younger plants. No such activity is detectable in the shoot tip and in internode regions.

Local activity of the *Sho* antisense promoter in flower buds (Figure 2a) and nodes (Figure 3) was also detectable in tobacco marker lines transformed with the PR2–GUS construct. In developing seedlings grown in tissue culture, the first promoter-specific GUS staining occurred in the central stem region when plants reached the six-leaf stage (Figure 2b). To test the influence of hormones on the activity of the *Sho* antisense promoter, we cultured young seedlings on the gibberellin GA3, the auxin IAA and three cytokinins. There was no direct effect of any of the hormones, as treatment for several hours did not influence the activity of the antisense promoter. Instead, we observed a long-term effect in cytokinin-treated plants, which was detected after hormone application had caused cell type de-differentiation. After several weeks of CK application, the root region had converted into a callus-like cell mass with enhanced PR2–GUS activity, which could also extend into the aerial parts (Figure 2b,c). In most lines, this effect was visible after 4–5 weeks (Figure 2c), but in one line we observed a more pronounced response after only 3 weeks. The common feature in all lines was the change in cell type, which appeared to be a prerequisite for PR2 activation.

**Figure 4:**
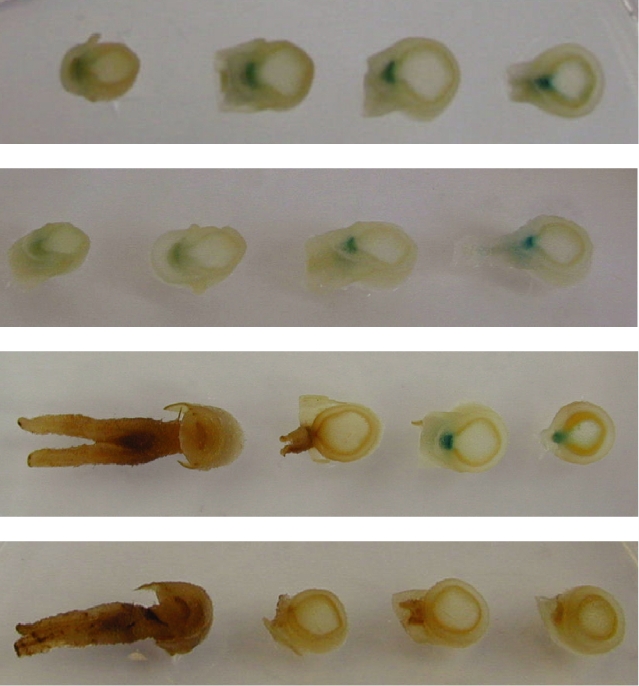
Response of *Sho* antisense promoter activity to shoot tip removal. Transverse stem sections of the top four nodes are shown for two control plants (top) and for two plants 1 week after the shoot tip had been removed (bottom). PR2–GUS activity is reduced in nodes where loss of apical dominance has induced side shoot growth.

The most pronounced example of localized activity of the antisense promoter was found in the nodal stem tissue around the base of axillary buds (Figure 3). In pea, auxins negatively regulate local CK biosynthesis in the nodal stem via repression of genes encoding two *Sho* homologues, *PsIPT1* and *PsIPT2* (Tanaka *et al.*, 2006). We therefore wondered whether the specific activity of the *Sho* antisense promoter in nodal stem tissue was an effect that augmented the negative regulation of CK production. As the release of auxin repression after decapitation reactivates *PsIPT* activity in pea, we tested whether decapitation also influenced the activity of the *Sho* antisense promoter. Similar to the hormone application tests, we did not observe any direct effect of decapitation on antisense promoter activity. One week after decapitation, however, we detected a reduction of antisense promoter activity in the upper nodes of a PR2–GUS transformant, from which new axillary shoots had emerged (Figure 4). Again, it appears that a change in cell type can alter the activation level of the antisense promoter.

We only detected GUS-specific staining in antisense promoter lines. RT-PCR analysis revealed that both the sense and the antisense promoter were universally active in seedlings, although at levels too low to be detectable by GUS staining (Figure S3).

### Detection of two types of Sho-specific small RNAs

**Figure 5:**
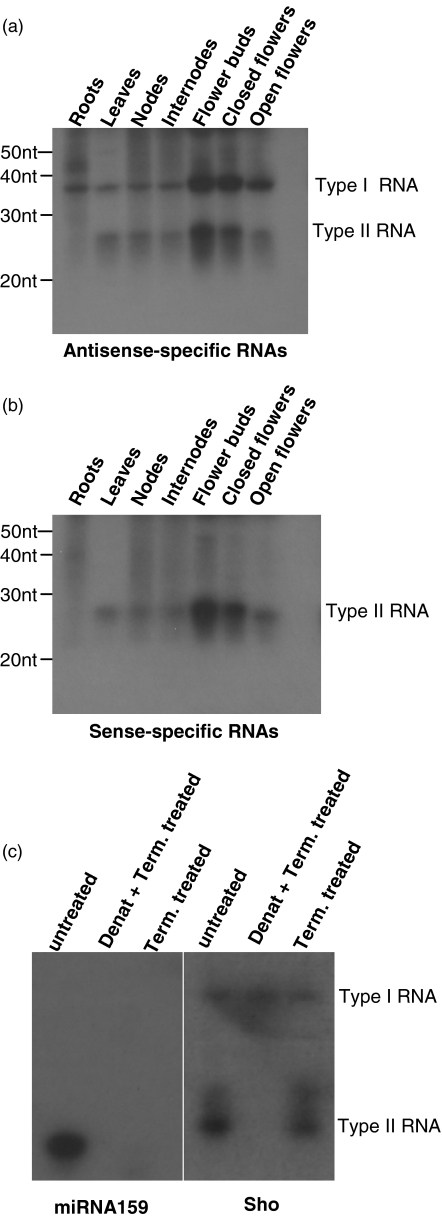
Hybridization with strand-specific riboprobes reveals two types of *Sho* specific small RNAs. (a) A *Sho* sense probe hybridizes to two types of RNAs, which are present in all tissues, except roots, where only the larger type I RNA is found. (b) A *Sho* antisense probe hybridizes to type II RNAs, which are present in all tissues except roots. (c) The type I RNA is resistant to terminator exonuclease that preferentially degrades substrates with a single 5′ phosphate. The type II RNA is sensitive to terminator exonuclease when samples were denatured for 5 min prior to treatment. As a control for efficient terminator activity, we used Arabidopsis microRNA 159, which is also present in petunia.

If antisense transcription leads to the formation of dsRNA that becomes a target for degradation, dsRNAs and small RNAs should be detectable. To examine the potential formation of dsRNA, we conducted an RT-PCR analysis of various tissues, following the treatment of the samples with RNAseONE, which selectively degrades single-stranded RNAs. An RNA preparation from flower buds, which contained relatively high levels of *Sho* antisense transcripts, also contained *Sho-*specific RNAs that were resistant to RNAseONE treatment (Figure S4), indicative of the presence of dsRNA. Hybridization of sense- and antisense-specific *Sho* probes to RNA samples enriched for small RNA molecules revealed two types of small RNAs. The type I RNA was antisense-specific, and was found in all tested tissues, with the highest representation in flower buds (Figure 5a). A smaller type II RNA represents both sense and antisense sequences, and was found in all tested tissues except roots (Figure 5a,b). We also tested the susceptibility of the two small RNA types to terminator exonuclease, which predominantly digests substrates with a 5′ monophosphate. The type I RNA pool was resistant against terminator treatment (Figure 5c), which suggests that the RNA 5′ ends contain di- or triphosphates. After denaturation, the type II RNA pool was sensitive to terminator exonuclease, indicative of 5′ monophosphate RNAs as they are produced by DICER cleavage.

**Figure 6:**
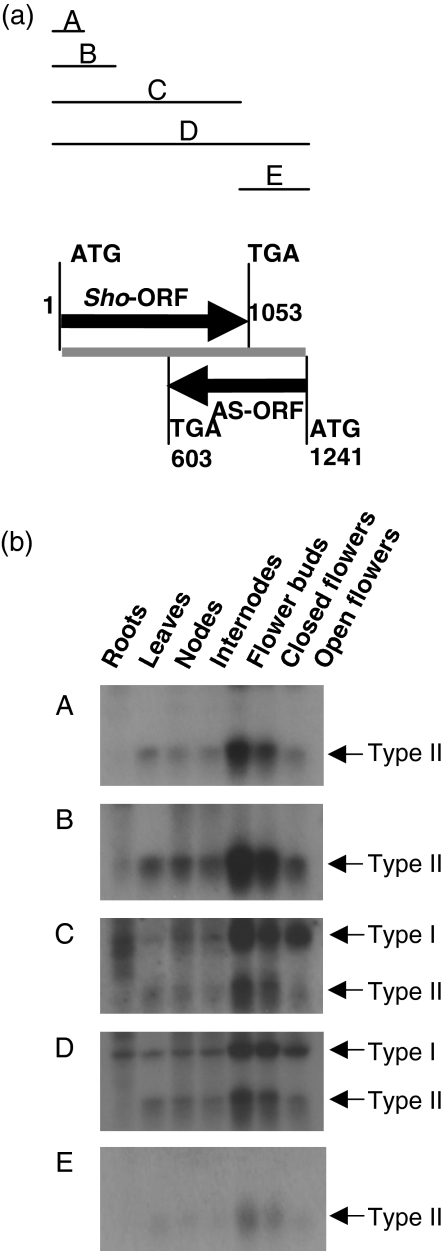
Distribution of type I and II small RNAs within the *Sho* locus. (a) Schematic picture of the *Sho* locus and the five probes A–E used for hybridization to small RNAs. (b) Northern blots of small RNA fractions hybridized to probes A–E. Type I small RNAs are detected by probes C and D, which include the central dsRNA region. Type II small RNAs are detected by all probes but are far less abundant in the 3′ region. Signals with probe E only became visible after the exposure time was increased fivefold compared with the other filters.

To determine the size of the two RNA types, we isolated RNA from the gel regions that contained the two types of RNA, and cloned and sequenced a random collection of clones (Appendix S1). Sequence analysis showed that the type I RNAs localized in a region that contains predominantly 35 nt long RNAs, and that the type II RNAs co-migrate with a pool that predominantly contains 24 nt long RNAs (Table 1). It therefore appears that all tissues tested contain an antisense-specific antisense RNA that is approximately 35 nt long, accompanied in most tissues by a pool of sense and antisense RNAs that are approximately 24 nt long. The latter RNA type, however, is not detectable in roots, which are the main source of CK supply. To examine the origin of the two small RNA types, we hybridized them to antisense-specific probes from various *Sho* regions (Figure 6). The 35 nt type II RNA was only detected by probes spanning the central overlapping region between the sense and antisense transcript. In contrast, the 24 nt pool hybridized to all probes with comparable intensity, except for the 3′ region of the *Sho* transcript, for which the signal strength was significantly reduced.

**Table 1:** Size distribution of randomly sequenced small RNAs that co-migrate with type I and type II RNA

Size (nt)	30	31	32	33	34	35	36	37	38	40
No. RNAs co-migrating with type I RNA	1	3	2	6	7	11	6	6	2	2
Size (nt)	21	22	23	24	25	26	27	28	29	30
No. RNAs co-migrating with type II RNA	4	5	14	47	0	2	1	0	0	0

## Discussion

Cytokinins are essential hormones for plant growth and development (Mok, 1994), and also for carbon transport and metabolism (Roitsch and Ehness, 2000). Local CK levels are determined by synthesis, transport and metabolism of CKs, as well as CK conjugation and CK release from conjugates (Mok *et al.*, 2000). Arabidopsis contains seven ATP/ADP isopentenyltransferase (IPT) genes that encode proteins for the rate-limiting step in CK synthesis. The promoters of four of these genes are suppressed by CKs, indicative of negative feedback regulation (Miyawaki *et al.*, 2004). CKs work in a complex interplay with auxins, which often act antagonistically by repressing CK synthesis (Takei *et al.*, 2004), but auxin-mediated activation has also been described for individual CK synthesis genes (Miyawaki *et al.*, 2004).

In *Petunia hybrida*, the *Sho* gene is the only known example of an IPT-encoding gene. The presence of a natural antisense transcript (NAT) of the *Sho* gene suggested its potential role as a regulator of the *Sho* transcript. So far, only two examples of NAT-specific regulation in plants have been identified that involve the production of small RNAs (nat-siRNAs). Degradation of the P5CDH transcript after salt induction of its NAT SRO5 requires DCL1 and DCL2, two members of the RNase III family of nucleases that specifically cleave dsRNAs, the RNA-dependent RNA polymerase RDR6, as well as SGS3 and NRPD1A. In a two-stage process, a 24 nt siRNA is formed by a biogenesis pathway dependent on DCL2, RDR6, SGS3 and NRPD1A, which establishes a phase for the generation of 21 nt siRNAs by DCL1 and for further cleavage of P5CDH transcripts (Borsani *et al.*, 2005). Generation of an endogenous siRNA of approximately 22 nt, nat-siRNA ATGB2, induced by *Pseudomon****a****s syringae*, also requires RDR6, NRPD1A and SGS3, but only one DICER enzyme, DCL1 (Katiyar-Agarwal *et al.*, 2006). These examples imply that some NATs can trigger a dsRNA degradation pathway that involves various RdRP and DICER functions producing specific small RNAs.

**Figure 7:**
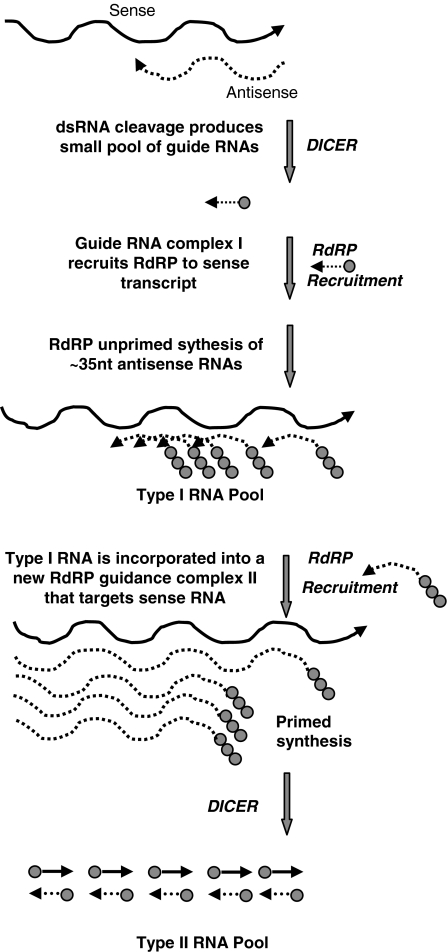
A hypothetical model for the production of type I and II RNAs. *Sho* sense and antisense transcripts form a partial double-stranded RNA target that regulates unprimed synthesis of the pool of type I RNA of approximately 35 nt by an RdRP activity. The antisense-specific type I RNAs can act as primer for the *Sho* sense transcript, generating dsRNA that are targeted by a DICER complex for degradation into type II RNAs of approximately 24 nt. This is discussed in more detail in the text. Filled circles illustrate 5′ phosphates.

In accordance with this mechanism, we detect *Sho*-specific sense and antisense transcripts, dsRNAs and two types of small RNAs. The resistance of the larger type I RNA pool to terminator exonuclease suggests that its RNAs do not contain monophosphates at their 5′ terminus. In *Caenorhabditis elegans*, terminator-resistant small RNAs have been found, which encode antisense fragments for mRNA transcripts, and it has been suggested that they represent triphosphorylated RdRP transcripts (Pak and Fire, 2007). While any comparison between *C. elegans* and petunia is difficult, it is conceivable that the modified 5′ terminus of the *Sho*-specific small RNAs reflects the involvement of specific RdRP complexes that are guided to NAT dsRNAs. We therefore propose the following working model (Figure 7): *Sho* sense and antisense transcripts associate in a dsRNA that is recognized by a DICER activity that produces a small pool of guide RNAs. These recruit an RdRP complex to *Sho* sense transcripts that serve as a template for non-primed synthesis of the pool of type I RNA of approximately 35 nt. As a next step, we propose that the type I antisense RNA acts as a primer for dsRNA synthesis using *Sho* sense transcripts as templates. The resulting dsRNAs would all cover the 5′ region of the *Sho* transcript but would differ with respect to coverage of the 3′ region, depending on where dsRNA synthesis was initiated. The dsRNAs serve as substrates for a DICER complex producing type II RNA breakdown products that are approximately 24 nt long. This model is in accordance with our observation that type II RNAs are under-represented at the 3′ end of the *Sho* transcript.

Both small RNA types were found in the various tissues tested, with the exception of roots, where only the type I RNA is detectable. It is unlikely that the lack of type II RNAs in roots is simply a consequence of low type I RNA levels, as we found comparable type I RNA levels in roots and in several other tissues that all contain type II RNAs (Figure 5). It therefore appears that the efficiency of type II RNA production is specifically inhibited or at least reduced in roots, which could be part of a tissue-specific adaptation in roots that maintains CK synthesis. Such an effect could, for example, be due to inefficient dsRNA synthesis, or to limited activity or access of the DICER complex in roots.

Our model allows at least two options for the control of local *Sho* transcript levels. The presence of the approximately 35 nt antisense primer could ensure that an increase in sense transcripts would also generate an increase in binding partners for the antisense primer, resulting in a proportional increase in RdRP-based antisense transcripts. This would explain why we find corresponding proportional levels of sense and antisense transcripts in many tissue types. Each cell type may have a different threshold level that ultimately determines the breakdown of dsRNA, and this threshold may be very high in roots, the only tissue where the approximately 24 nt RNA type is not found.

A second option to modulate the local efficiency of the NAT pathway is activation of the antisense promoter in specific cell types, which would improve sense–antisense pairing and increase the pool of approximately 35 nt primers. An important factor for the tissue-specific adjustment of NAT synthesis could be the ability to sense local CK concentrations, as two of our experiments suggest. The application of external CKs induced changes in root tissue that reorganized into callus-like tissue (Figure 2b). At the same time, the activity of the antisense promoter was enhanced. We interpret this as the consequence of the cell type change and accumulation of high CK levels, which stimulates the NAT system to inhibit local CK synthesis. Similarly, the remaining local activity of the antisense promoter at the base of axillary buds (Figure 3) could reflect a cell-specific enhancement of the NAT system in an auxiliary role to maintain apical dominance. There are, however, no indications that local activity of the antisense promoter would be sufficient to maintain apical dominance, as decapitation triggers axillary bud growth of side shoots. Mechanistic models for apical dominance focus on the effects of auxins on auxin transport out of the branches and on the repression of CK synthesis in axillary buds (Leyser, 2005). The later effect has been demonstrated in pea, where auxins control CK synthesis in the nodal stem, repressing the promoter activity of two *PsIPT* genes. After decapitation, this block is released, and CK synthesis in nodal stems facilitates outgrowth of axillary buds (Tanaka *et al.*, 2006). In the newly formed axillary shoots that develop after decapitation, the tissue that was located at the base of the axillary bud has been reorganized and has lost its specific competence for enhanced activity of the *Sho* antisense promoter.

The synthesis of CKs is only one element in the complex network that determines local levels of biologically active CKs. Its regulation has largely been discussed with regard to promoter specificity and response. The detection of antisense transcripts with the potential to mediate transcript degradation introduces an additional tool for CK control and adaptation. The involvement of small RNAs offers options for transitive silencing of secondary targets (Bleys *et al.*, 2006), for the cell-to-cell spread of silencing signals over short distances (Dunoyer *et al.*, 2005), or for their long-range transport via the vascular system (Palauqui *et al.*, 1997). It would therefore be interesting to test whether these features play a role in the regulation of gene family members and in the establishment of CK gradients.

## Experimental procedures

### Construct design

The promoterless GUS construct was based on cloning a *Bam*HI–*Eco*RI fragment containing a GUS marker gene and a nopaline synthase (NOS) polyA region from pBI101 (Clontech, http://www.clontech.com/) into the *Bam*HI–*Eco*RI site of pGreen 0029 (Hellens *et al.*, 2000). The genomic region of the *Sho* sense gene promoter was amplified using primers PR1-R-*Bam*HI (CGGGATCCACGGAAAACATGACAAATGGTAG) and PR1-F-*Xba*I (GCTCTAGAAGCATAGTTGGATTAACGGTAC). After digestion with *Bam*HI and *Xba*I, the PCR fragment was inserted into the promoterless GUS construct digested with *Bam*HI and *Xba*I. The *Sho* antisense promoter region was amplified using primers PR2-F-*Pst*I (ATTGGTTCTGCAGCTTGTATTAACATAGAACCTGA) and PR2-R-*Bam*HI (CGGGATCCGAAATATGAAAGAAATACTTATCAAG). The PCR product was digested with *Pst*I, filled in with Klenow enzyme and digested with *Bam*HI, before being inserted into a promoterless GUS construct that had been digested with *Xba*I, filled in with Klenow enzyme and digested with *Bam*HI. Deletion constructs of the antisense promoter region were obtained by *Bsu*361 digestion and *Bal*-31 exonuclease treatment.

### Plant transformation and GUS analysis

For plant transformation, constructs were transformed into *Agrobacterium tumefaciens* GV3101 (pMP90) by electroporation. The generation of transgenic lines in *Nicotiana tabacum* cv. SRI Petit Havana and the isolation of protoplasts for transient assays have been described previously (Zubko *et al.*, 2002). Protoplasts were transformed using Mg^2+^/PEG solutions according to the method described by Fehér *et al.* (1991). Three independent transformation experiments were performed for each construct using 25 μg of plasmid DNA. As a control for transformation efficiency, 2 μg of the pJIT53 35S-luciferase construct (JIT catalogue, John Innes Centre, Norwich, UK; http://www.pgreen.ac.uk) was used in each experiment.

GUS staining (Jefferson, 1987) was performed on plants grown in the greenhouse at 24°C with 16 h day light. Decapitation of transgenic plants was performed when six nodes had formed. To test the effect of hormones on antisense promoter activity, transgenic seedlings expressing the PR2–GUS construct were grown on MS medium supplied with 1 mg l^−1^ of gibberellic acid (GA3), indole-3-acetic acid (IAA), 6-benzylaminopurine (BAP), isopentenyladenosine (iPA) or zeatin riboside (Z). Quantitative measurement of GUS activity was performed on three replicates. The data were standardized to the corresponding LUC activity. Extraction and analysis of luciferase activity were performed as described previously (Luehrsen *et al.*, 1992).

### RNA analysis

Plant material was collected from leaves, stem, stem nodes and roots of greenhouse plants grown in soil for 6 weeks, as well as from flowers, flower buds and sepals of flowering plants. RNA extraction and cDNA synthesis were performed as described previously (Zubko *et al.*, 2002). For strand-specific cDNA, we used 2 μg RNA and the primers Sho-R-plus (TCGACCGCAATCGTGCTTCGAGCCTCAGTCCAACAAAAAACGGTTCAC) for sense strand synthesis and ORF2-plus (TCGACCGCAATCGTGCTTCGAGCTACAACTGTTGTTTTATTTGGGTCG) for antisense strand synthesis. Lower-case letters indicate (‘plus’) sequences that are not present in the *Sho* gene. For each sample, 2 μl of cDNA was used in PCR reactions. Primers 704F (ATGTTAATTGTAGTACATATTATTAGC) and 704R (TCAGTCCAACAAAAAACGGTTCAC) or 704F and ‘plus’ (TCGACCGCAATCGTGCTTCGAGC) were used to amplify the sense transcript; amplification primers for the antisense transcripts were ORF2R (TACAACTGTTGTTTTATTTGGGTCG) and ORF2F (ATGTATTTATGTCTCAAAAAAAAAATTGC) or ORF2F and ‘plus’. PCR conditions were one cycle at 94°C for 2 min, 25 cycles at 94°C for 1 min, 60°C for 1 min and 72°C for 1 min, and 72°C for 5 min. PCR products were separated on a 1% agarose gel, blotted on Hybond+ membrane (Amersham Bioscience; http://www.amersham.com) and hybridized with a ^32^P-labelled *Sho* probe at 65°C according to the method described by Koes *et al.* (1987). For polyA site mapping, cDNA was prepared from 2 μg of RNA using an oligo(dT) primer.

For PCR, an oligo(dT)–*Eco*RI primer and *Sho*-gene specific primer AF2 (ACATGTCGTCATCCACTGTAGTAA) were used. Amplified fragments were digested with *Eco*RI and *Bam*HI, and cloned into pBluescript SK (Stratagene; http://www.stratagene.com) for sequencing. To identify polyA ends of antisense transcripts, RT-PCR was performed using an oligo(dT)–*Eco*RI primer and primer ORF2F. PCR products were separated on a gel and hybridized as described above.

For dsRNA analysis, RNA was incubated with RNaseONE (Promega, http://www.promega.com/) for 30 min at 37°C. The remaining dsRNA was precipitated, dissolved in water, and denatured for 10 min at 95°C before cDNA was prepared using an oligo(dT) primer and the primer *Sho*-R-plus that is located in the sense–antisense overlapping region. For PCR, primer ‘plus’ and primer AF3 (CAGGTTTTCGGATCCGGGTTTGGAAC) were used. PCR conditions were: one cycle at 94°C for 3 min, 40 cycles at 94°C for 30 sec, 60°C for 30 sec and 72°C for 30 sec, and finally 72°C for 5 min.

Small RNA isolation and hybridization were performed as described previously (Hamilton and Baulcombe, 1999). Various parts of the *Sho* region were amplified and cloned into pCR4-TOPO (Invitrogen, http://www.invitrogen.com/). Riboprobes were transcribed by T7 or T3 polymerase (Promega) according to the manufacturer’s recommendation. Before hybridization, the probes were hydrolysed to a length of approximately 50 nt using an alkaline hydrolysis buffer. Hybridization was performed overnight at 42°C. Filters were washed twice at 45–55°C for 30 min in 2× SSC, 0.5× SDS, and twice for 15–20 min in 2× SSC, 0.2× SDS. The RNA Decade™ marker (Ambion; http://www.ambion.com) was use as a size reference.

Cloning and sequencing of small RNAs were performed according to the protocol described by D. Baulcombe, which is available on the Sainsbury laboratory website (http://www.tsl.ac.uk/dcb/services/Small_RNA_cloning_protocol.pdf). To check for terminal phosphates, small RNAs were treated with terminator 5′-phosphate-dependent exonuclease (Epicentre Biotechnologies; http://www.epibio.com) according to the manufacturer’s recommendations, with or without denaturation of the sample at 95°C for 5 min.

## Supplementary material

The following supplementary material is available for this article online:

Figure S1 Deletion analysis of the Sho antisense promoter.

Figure S2 Mapping of polyadenylation sites of the antisense transcript.

Figure S3 Sho sense and antisense promoter expression in transgenic reporter lines.

Figure S4. Removal of ssRNA by RNAseONE treatment.

Appendix S1. Sequence of randomly selected small RNAs listed in Table 1.

This material is available as part of the online article from http://www.blackwell-synergy.com.
