# Supplementary figures and images for: A natural antisense transcript of the Petunia hybrida Sho gene suggests a role for an antisense mechanism in cytokinin regulation
Source: Plant J. 2007 Dec;52(6):1131–9. doi: 10.1111/j.1365-313X.2007.03309.x (PMC2253869; doi:10.1111/j.1365-313X.2007.03309.x)

**a**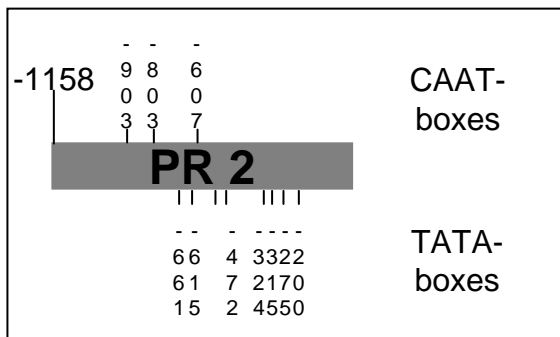**b**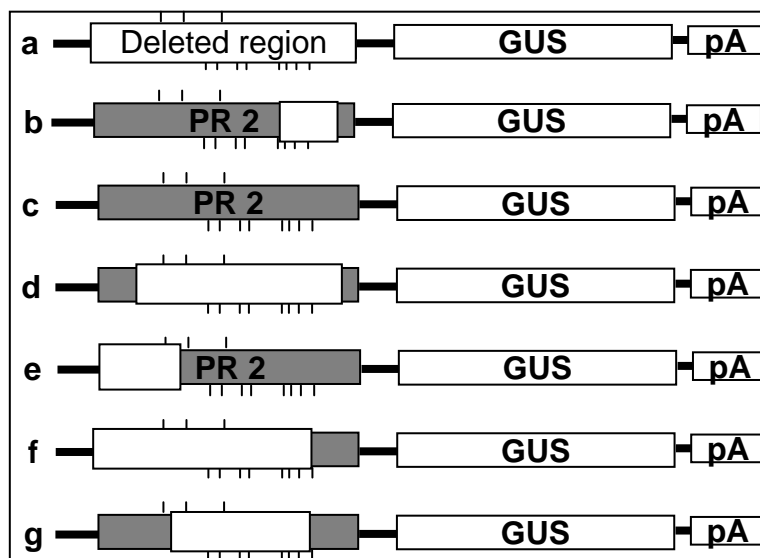**c**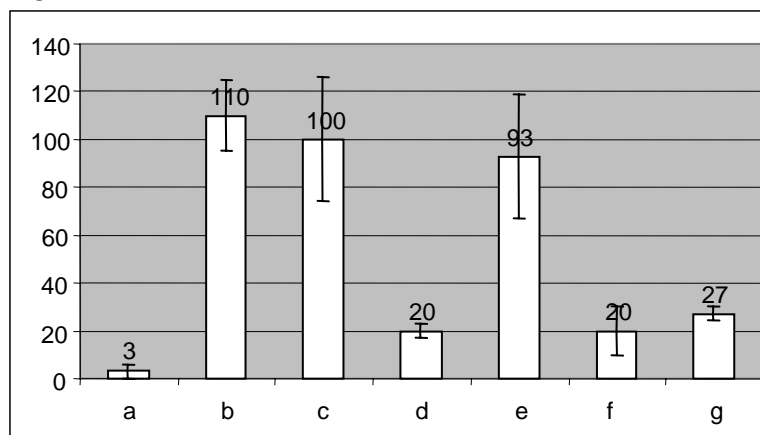

Supplement: Figure S1 — Deletion analysis of the Sho antisense promoter. [file tpj0052-1131-FigS1.pdf]

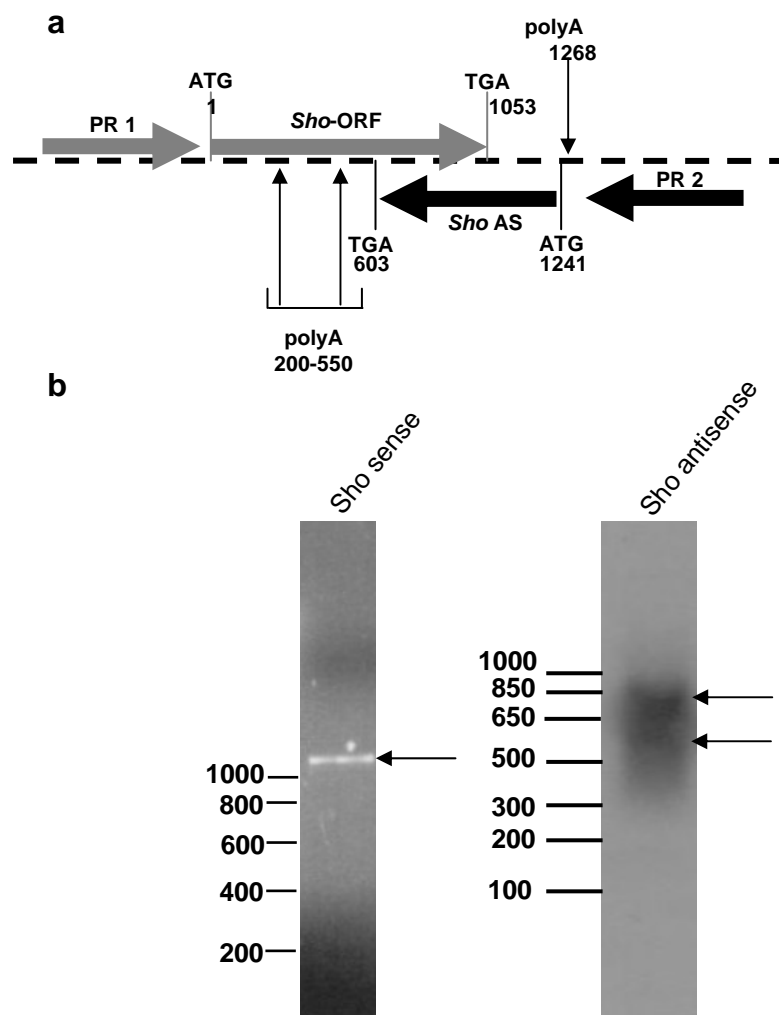

Supplement: Figure S2 — Mapping of polyadenylation sites of the antisense transcript. [file tpj0052-1131-FigS2.pdf]

**a**

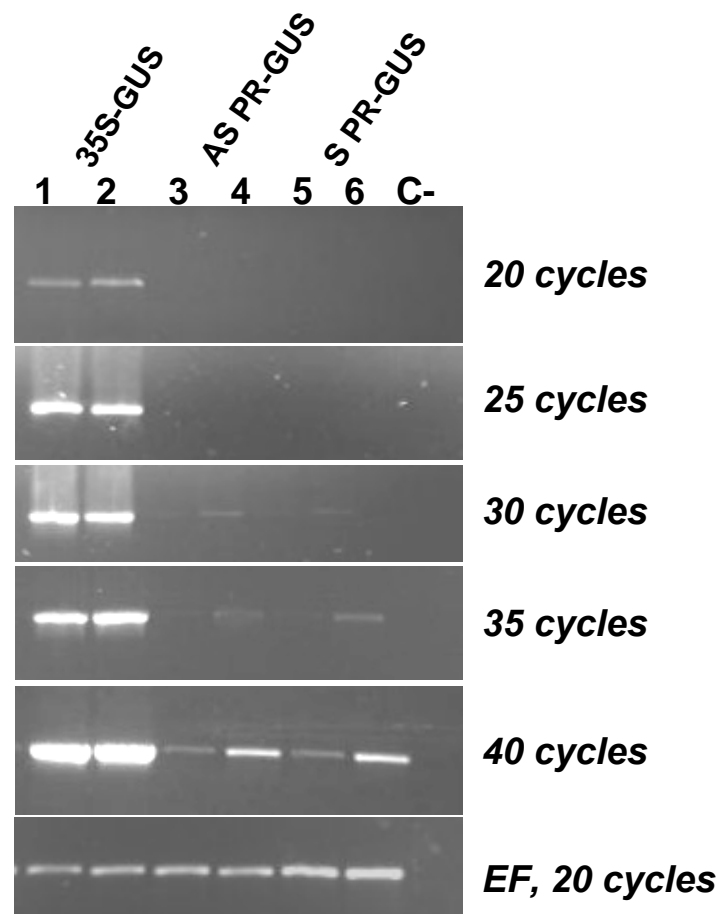

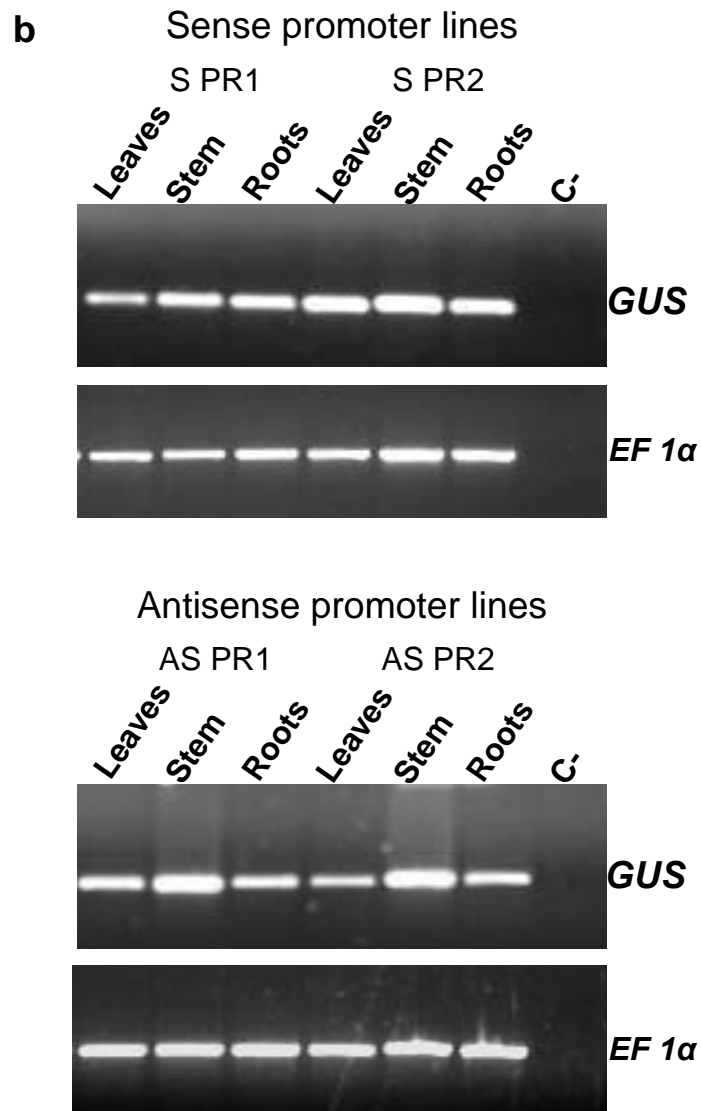

Supplement: Figure S3 — Sho sense and antisense promoter expression in transgenic reporter lines. [file tpj0052-1131-FigS3.pdf]

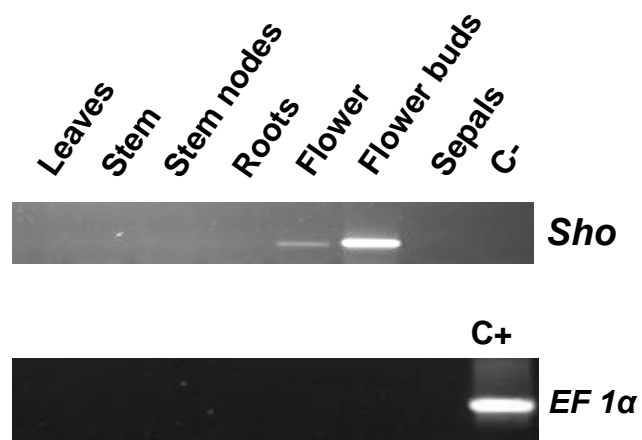

Supplement: Figure S4. — Removal of ssRNA by RNAseONE treatment. [file tpj0052-1131-FigS4.pdf]
