# Supplementary material for: A natural antisense transcript of the Petunia hybrida Sho gene suggests a role for an antisense mechanism in cytokinin regulation
Source: Plant J. 2007 Dec;52(6):1131–9. doi: 10.1111/j.1365-313X.2007.03309.x (PMC2253869; doi:10.1111/j.1365-313X.2007.03309.x)
Supplement: Appendix S1. — Sequence of randomly selected small RNAs listed in Table 1. [file tpj0052-1131-tableS1.doc]

**Supplementary table 1: Sequence of randomly selected small RNAs listed in table 1**

**Small RNAs co-segregating with the type II RNA pool**

1. GGGGTACAAAGTGGATCTAAT – 21

2. TGGTGGAGGTGGTGGTTGTGGC - 22

3. GGGATAGACTGGGTATGTTGTTGT – 24

4. GGGTATACTGGGTATGTTGTTGT – 23

5. CTGGGTATGTTGTTGTTACAAGC – 23

6. GGGATAGACTGGGTATGTTGTTGT -24

7. AGACTGCACTGGGTATGTTGTTGT – 24

8. AGGATACACTGGGTATGTTGTTGT – 24

9. CTGGGTATGTTGTTGTTGTTAACC – 24

10. CGGCAACAACAACATACCCAGTGT – 24

11. GAAAACAACAACAACATACCATG – 23

12. GTCACTGGGTATGTTGTTGTTGTA – 24

13. GGGATACACTGGGTATGTTGTTGT -24

14. TGGACACACTGGGTATGTTGTTGT – 24

15. GGGATATACTGGGTATGTTGTTGT – 24

16. GCAGGTGTCAGCATCAGTGTCTTC – 24

17. AGGTGTCAGCATCAGTGTCTTC - 22

18. GCAGGTGTCAGCATCAGTGTTTTT – 24

19. ATGTGAAGGAGTTTGCCTGGGCAC -24

20. TCTTGGACCTCCTCATCTCAG – 21

21. CATTGTTCATGGACTTCCTCCTCC – 24

22. GGCGGGTAGGTTGGTGGAGGA – 21

23. GGCGGCACTTCAAGCTATCTGGGG – 24

24. AGTCTGAGTGAAATATACGCAGAA – 24

25. CCGGTGTCCGGTATCCTCATGGCC – 24

26. CCGTGTAGGCTGCATCCATCACAT – 24

27. GCATTCATCCCATCTCATCGCACC – 24

28. TTATGGGTAGAGGTGATGCGAAAC – 24

29. GATTATGATTAAGGGTGAAGGGAT – 24

30. TCAAATCCCCTACAAACCTGCATA – 24

31. CAGACCACATGGAAGACAGGGTT – 23

32. CAGACCACATGGAAGACAGGGTT – 23

33. GGGTTAAGTGGAAGAAGACATGT – 23

34. CTTGAAAAAACAAAAACCAAATGGTC – 26

35. AAAAAAAATAAAACCAAAATCAAC – 24

36. CACCAAGGTGAGGTCTAGGGAGAG – 24

37. TCCAAATTTGCAAAGGTCCTTCCT – 24

38. AAGACCAAAGCTCTAAACATGGCC – 24

39. TGATCAGGGATAGAATCCGAGGT – 23

40. ATGATCAGGGATAGAATCCGAGGT – 24

41. TGATCAGGGATAGAATCCGAGGT – 23

42. AGAAATTGAGGATACCCGGTGGAT – 24

43. GGTTGAGGATTTGGATAGACGGTC – 24

44. TAGGGGGTTTACGGGTCCGGGTCC – 24

45. AGGGGGTTTACGGGTCCGGGTCCG – 24

46. GGGGTTTACGGGTCCGGGTCCGA - 23

47. AGGGGTTTACGGGTCCGGGTCCG – 23

48. TTAGGGGGTTTACGGGTCCGGGT – 23

49. TAGGGGTTTACGGGTCCGGGTC - 22

50. AGCAGAAAACACGTTGTCGGTTC – 23

51. TAGGGGGTTTACGGGTCCGGGTCC – 24

52. AGGGGTTTACGGGTCCGGGTCTGG – 24

53. TAGGGGGTTTACGGGTCCGGGTAC – 24

54. CAAACAGGTATACGGGTCCGGGTT – 24

55. TGGCCGGGTCGTGCCTCCGGC – 21

56. CCAGTGAGGAAAGGGATCGGGGTA – 24

57. AGGAAGAGGTGTGCGTATGAATGG – 24

58. AAGAGGTGTGCGTATGAATGGA – 22

59. TGGAGTTGTTGTGAGGAGCAGGGT – 24

60. AGGTGAGGGAAGTAGGGTGGGTT – 23

61. ACAAGCAGGGGCGGACCTAGGTA – 23

62. AGTAATAAAGAGGAAAAAAGGAAAAAA – 27

63. CACGCCTAAGGAGGAGAAGGGAAG – 24

64. CGCCTAAGGAGGAGAAGGGAAGCC – 24

65. AAGTAGGGGGACCAAGTTGCAGAC – 24

66. TCGAACTTGTCACTTGGTACCACC – 24

67. ACCAAGCTATTGCTAGCTCAAGCA – 24

68. AGTACTCATTATTTTATTTATGGGAC – 26

69. GGAAAACATAGAAGAAAAAGGTCA – 24

70. TGATGAATAGTGAGTTGTGAGG – 22

71. GTGGGGAAATACATGGAATTTGAA – 24

72. ACGGGTATACGGGTCCGGGTTCGG - 24

73. TCGTGTGCCCGAACAATACCCAGA – 24

**Small RNAs co-segregating with the type I RNA pool**

74. TCCTCAGATAGCTCAGATGGTAGAGCGGTCGGCG - 34

75. CAAAATGTCACGGGTTCAAATCCTGTCATCCCTACC - 36

76. CGGATGATGGAACGGTGTTGAGCCGGTCCGCCGAT - 35

77. TGCGAGAGGTCCGGAGTTCGTTTCTCCGAATGCCC - 35

78. CCGAGAGACCCGGGTTCAGGTCCCGGCAACGGAACCA - 37

79. TGCGAGAGGTCCCGAGTTCATTTCTCGGAATGCCC - 35

80. TGCGAGAGGTCCCGAGTTCATTTCTCGGAATGCCC - 35

81. CGGGTGACCCGGGTTCGTTCCCCGGCAACGGCGCCA - 36

82. GCGAGAGGTCCCGAGTTCGTTTCTCGGAATGCCCC - 35

83. CCGAGAGACCCGGGTTCAGGTCCCGGCAACGGAAC - 35

84. TCCGTTGTAGTCTAGGTGGTTAGGATACTCGGC - 33

85. GCGAGAGGTCCCGAGTTCGTTTCTCGGAATGCCC - 34

86. GCAATAGACCCGGGTTCGGCTCCCGGCAGACGC - 33

87. GAGAGTAGTACTGGAATGGGTGACCCTCCGGGAAGTCCTC - 40

88. GNGAGGCTCTGGTTCAAGTCCAGGATGGCCCACC - 34

89. GCGAGTCTCTGGTTCAAGTCCAGGATGGCCCACCA - 35

90. ACGAGAGGTCCCAAGTTCATTTCTCGGAATGCCC - 34

91. GCTCTCGTAGCTCAGTTGGTTAGAGCACCCGTTT - 34

92. TGCGAGAGGTCCGGAGTTCGTTTCTCCGAATGCCC - 35

93. CCGAGAGACCCGGGTTCAGGTCCCGGCAACGGAACCA - 37

94. CACCATGCGCGGGTTCAATTCCCGTCGTTCGCCCC - 35

95. GGTACAGACCCGGGTTCGTTTCCCGGCTGGTGC - 33

96. TGAGAGGTCCCGAGCTTCGTTTCTCGGAATGCCCCCC - 37

97. GCGAGGTCTCTGGTTCAAGTCCAGGATGGCCCACCA - 36

98. GCGAGAGGTCCCGAGTTCATTTCTCGGAATGCCCCCCA - 38

99. GCAATAGACCCGGGTTCTCCCGGCAGACGC - 30

100. GTCGTTGTAGTATAGTGGTAAGTATTCCCGC - 31

101. CGAGAGGTCCCGAGTTCATTTCTCGGAATGCC - 32

102. TCCGTTGTAGTCTAGGTGGTTAGGATACTCGGC - 33

103. TCCTCAGTAGCTCAGTGGTAGAGCGGTCGGC - 31

104. GTCGTTGTAGTATAGTGGTAAGTATTCCCGC - 31

105. GATTGGTCGTAGGTTCGAATCCTACTTGGGGAGCCA - 36

106. GCGGGTGACCCGGGTTCGATCCCCGGCAACGGCGC - 35

107. CGCGGGTGACCCGGGTTCGGTCCCCGGCAACGGCGTCA - 38

108. GCGGGTGACCCGGGTTCGATCCCCGGCAACGGCGCC - 36

109. CAGGAGACCCGGGTTCGTTTCCCGGTAACGGAA - 33

110. CGGGACGTGGCGGTTGACGGCTTTCTATCCAT - 32

111 ATCCACAGGATGTGAACCTTTATACCTATAAGGCG - 35

112. CCGAGAGACCCGGGTTCATGTCCCGGCAACGGAACCA - 37

113. GCCCTTTTAACTCAGTGGTAGAGTAACGCCATGGTAAGGC - 40

114. GCGAGAGGTCCCGAGTTCGTTTCTCGGAACGCCCCC - 36

115. GCAATAGACCCGGGTTCGGCTCCCGGCAGACGCA - 34

116. ACGGGTGACCCGGGTTCGATCCCCGGCAACGGCGCCA - 37

117. GCGAGAGGTCCCGAGTTCATTTCTCGGAATGCCC - 34

118. TCCAGCGACCTGGGTTCGGCTCCCGGTAGGACCTCCA – 37

119. GCTGGAGTAGCTCAGTTGGTTAGAGCTTGTGGC - 33
