# Supplementary material for: A natural antisense transcript of the Petunia hybrida Sho gene suggests a role for an antisense mechanism in cytokinin regulation
Source: Plant J. 2007 Dec;52(6):1131–9. doi: 10.1111/j.1365-313X.2007.03309.x (PMC2253869; doi:10.1111/j.1365-313X.2007.03309.x)
Supplement: Supplementary file 6 [file tpj0052-1131-Fig_legends.doc]

**Supplementary figure 1: Deletion analysis of the *Sho* antisense promoter.**

(a) Map of the antisense promoter region with putative CCAT and TATA boxes marked. The numbers refer to the position of these elements, relative to the ATG site of the antisense transcript.

(b) Map of eight deletion constructs (a-g) of the antisense promoter linked to a GUS reporter construct. Deleted regions are indicated as white boxes. The position of the putative CAAT and TATA elements are indicated by black bars.

(c) Expression values for the eight deletion constructs in a transient assay system. Despite extensive deletions of different promoter regions, a minimum promoter activity of 20% is retained.

**Supplementary figure 2: Mapping of polyadenylation sites of the antisense transcript.**

(a) Map of the *Sho* region with polyadenylation positions, relative to position +1 at the start of the *Sho* ORF.

(b) RT-PCR products using a polyA-specific primer and a primer specific for the Sho sense and antisense transcript. The variable length of the a antisense-specific RT-PCR products indicates variable polyA sites.

**Supplementary figure 3: Sho sense and antisense promoter expression in transgenic reporter lines.**

(a) RT-PCR analysis of promoter-GUS transformants with different cycles, demonstrates the very low activity of the Sho sense (S PR-GUS) and antisense promoter (AS PR-GUS), compared to the 35S promoter (35-GUS).

(b) RT-PCR analysis in transgenic tobacco plants that contain a GUS reporter gene driven by the *Sho* sense promoter (S PR1 and S PR2) or antisense promoter (AS PR1 and AS PR2), shows comparable expression levels in different tissues.

Except for the local antisense promoter activity depicted in figures 2-4, promoter activities were too low to produce significant GUS-specific staining.

**Supplementary figure 4: Removal of ssRNA via RNAseONE treatment.**

RT-PCR following RNAseONE treatment indicates the presence of *Sho*-specific dsRNAs in flowers and flower buds, two tissues with enhanced antisense transcripts (Fig. 1c). An RT-PCR for an elongation factor gene *EF 1α* was used to determine the efficiency of RNAseONE treatment.
